# Supplementary material for: Are carbon emissions trading and green financial instruments synergistic? -Comprehensive quantitative research based on content analysis
Source: PLoS One. 2024 Mar 7;19(3):e0298601. doi: 10.1371/journal.pone.0298601 (PMC10919601; doi:10.1371/journal.pone.0298601)
Supplement: S1 Data — (ZIP) [file pone.0298601.s001.zip › Data/地级市Z_F工作报告.zip!/┴╔─■/▒╛╧¬╩╨/└┤╫╘╣1⁄2╓┌║┼í╛┬φ┐╦╩2╛▌═°í┐_2018.docx]

各位代表：

我代表市人民政府向大会作报告，请予审议，并请市政协委员和各位列席会议的同志提出意见和建议。

一、过去五年及2017年工作回顾

市十五届人大一次会议以来的五年，是本溪发展史上极不平凡、极具挑战的五年。面对经济发展新常态带来的深刻变化，在市委的坚强领导下，在市人大和市政协的监督支持下，市政府团结带领全市人民，全面贯彻党的十八大、十九大精神，以习近平新时代中国特色社会主义思想为指导，落实新发展理念和“四个着力”“三个推进”，坚持以供给侧结构性改革为主线，抢抓新一轮振兴机遇，攻坚克难、砥砺奋进，较好地完成了本届政府主要目标任务。

产业结构发生深刻变化，发展质量进一步提升。中药材等特色高效农业蓬勃发展，本溪辽五味、林下参等被评为全国地理标志登记保护产品，市级以上农业产业化龙头企业达125户。本钢冷轧高强钢竣工投产，本钢出口连续五年领跑全国钢企。钢铁深加工产业不断壮大，生物医药产业快速发展，高新区迈入国家高新区生物医药产业综合竞争力50强、列第28位。旅游总收入年均增长12%以上。新增金融机构16家、大型现代物流企业9户，金融机构贷款余额比2012年增长近90%。供给侧结构性改革“三去一降一补”任务全面落实。

产业园区发展优势凸显，创新活力进一步提升。建设省级经济开发区6个，入驻企业547户。高新区建设取得重大进展，集聚上海医药、上海绿谷、华润三九、天士力、日本卫材、韩国大熊制药等医药类项目126个，49户企业获得GMP、GSP认证，17户企业销售收入超亿元，3户医药企业实现新三板上市。建成10万平方米的创新园和创业园，引进华大基因等海内外高层次科研机构50家，构建8个省级公共服务平台，在研项目380项、完成转化147项。引入沈阳药科大学等6所医药类高校，有力增强了高新区创新发展活力和竞争力。

改革开放步伐持续加快，发展动力进一步提升。一批改革取得突破性进展，共取消、下放和调整行政职权1308项，率先在全省实现项目网上并联审批。深入实施营改增、资源税从价计征等税制改革，营改增累计减税2.4亿元。基本完成农村土地确权127万亩，集体林等试点改革成功经验全国推广。福耀玻璃、万达广场、同仁堂等一大批重大项目落户本溪，累计实际利用外资13亿元，实现出口867亿元，出口增速位居全省前列。沈本一体化加速推进，通信、交通等领域融合发展取得阶段性成果。

各项事业全面协调发展，群众获得感进一步提升。一大批惠民举措落地实施。城镇、农村常住居民人均可支配收入累计分别增长46%和53%。企业退休人员基本养老金人均增加826元。118908名贫困人口实现脱贫，55个贫困村销号，桓仁县贫困县摘帽，脱贫攻坚取得决定性成果。高考一本上线率、重点大学录取率始终保持全省领先。基层卫生医疗机构、公共文体设施均实现全覆盖。克服重重困难，保证了养老金按时足额发放，保障了城乡困难群体基本生活，有效防范政府债务、财政支出风险。平安本溪建设深入推进，社会大局和谐稳定。老旧小区、城中村、棚户区等改造面积达2000余万平方米，暖房子工程实现主城区全覆盖。沈丹客专建成通车，结束本溪无高铁历史。衍水大桥、千金岭隧道、峪龙隧道等一批重点项目竣工，打通城市东、南出口。生态环境质量持续改善，荣获全国绿色发展优秀城市、国家园林城市、省级环保模范城市等称号，国家森林城市建设通过复核。

各位代表，刚刚过去的2017年，是本届政府施政履职的收官之年，市政府统筹做好稳增长、促改革、调结构、惠民生、防风险各项工作，保持了经济社会持续健康发展。全市地区生产总值810.1亿元，增长2.5%；规模以上工业增加值增长1.7%；一般公共预算收入64.6亿元，增长16.2%；固定资产投资210.5亿元，下降10.4%；社会消费品零售总额372.1亿元，下降1.4%；出口178亿元，增长43.4%；城镇常住居民人均可支配收入31001元，增长6.4%；农村常住居民人均可支配收入14540元，增长7.1%。

一年来，主要做了以下工作：

（一）稳中求进抓发展，经济实现持续增长

抓存量，工业经济再上新台阶。深入落实领导干部包扶企业责任制。全力服务本钢发展，解决本钢冷轧高强钢建设等问题84项，支持本钢新增优质产能200万吨。充分发挥了本钢对地方企业的辐射作用，协调13户地方采选企业向本钢供货317万吨。华岳精工、金桥焊接、银龙预应力、聚鑫达、东铄新材料等钢铁深加工企业销售收入增长50%以上。新增2个销售收入超亿元医药单品种，华润三药、参源堂等医药企业销售收入、税收均增长20%以上。新增本溪湖、南芬两个省级经济开发区。增加规上工业企业33户，稳定运行企业达117户。

抓增量，项目建设再创新局面。落实全市领导干部联系100个重点项目制度，破解土地、资金、人才等难题，新增耕地开发271公顷，推动了379个项目加快实施。积极“走出去、请进来”，签约汇源田园综合体、天士力健康食品产业园、兴隆大家庭等重点项目78个。经过多年不懈努力，投资20亿元的福耀玻璃项目落户本溪，主体工程封顶。顺势产业大厦、祥云健康产业园、腾龙房车制造、宇航矿业等亿元以上重点项目开工建设。国家电投本溪热电、上药集团北方药业、好护士健康科技等重点项目相继竣工。

抓升级，服务业形成新态势。在旅游业方面，铁刹山、绿石谷晋升4A级景区，新增小市一庄、枫林城堡、韩家村、甬子峪等一批特色民宿，幸福花海、虎谷峡、东风湖温泉水世界推出新产品，康溪温泉理疗中心、枫香谷温泉会馆等项目投入运营。建成40处标准化旅游公厕。旅游营销效果明显，接待游客和旅游总收入均增长13%以上。在商贸流通业方面，“银亿汇”城市综合体实现试运营，万达广场、红星美凯龙等项目加快建设，华联社区连锁生鲜超市达21家。快递物流产业园入驻企业110户。建立村级电商服务站82家。

抓特色，农业实现新发展。调减玉米种植面积2.75万亩，发展刺五加、林下参等经济作物5.4万亩，建成东北首个“南菜北种”生产基地。组建本溪农村产权交易中心，流转土地2万亩，家庭农场达到77个。深度开发本溪县刺五加干混冲剂等系列产品。桓仁山参生态原产地产品保护通过国家认定。

抓机制，优化营商环境推出新举措。出台《本溪市优化营商环境实施方案》，明确了27条举措、“七个不允许”要求，建立实施营商环境绩效考核、企业评议政府部门等制度，省考核市场主体对营商环境满意度达到86.5%。严肃责任追究，开展专项整治行动88批次，查处典型案件138起，追责问责41人。

抓培育，创新发展取得新成果。9个平台获批省级产业专业技术创新平台，发布技术成果369项、转化35项，新增高新技术企业22户。加大品牌培育力度，全市获得省名牌产品10个，龙山泉啤酒公司成为省食品行业唯一获得“省长质量奖”企业。本钢不锈钢、硅钢、电镀锌等产品打入国际市场。

（二）多点突破促改革，发展活力显著增强

扎实推进供给侧结构性改革。狠抓“三去一降一补”任务落实，提前完成关闭煤矿17家、去产能97万吨工作目标。结合棚改去库存，货币化安置比例达96.2%，商品房去库存81.7万平方米，增长12.2%。重点国有企业杠杆率降到80%以下。协调36户企业享受省直供电政策，降成本1.5亿元。进一步补齐环境保护、民生保障等短板。

全力推动国资国企改革。整合成立11家市属、12家县区所属国有控股公司，本溪水洞旅游集团、新时代传媒等企业实现股权多元化，23户市属全民所有制企业完成公司制改制。驻溪央企、省属企业“三供一业”分离移交扎实推进。

继续深化“放管服”改革。完成第六轮简政放权任务，取消、下放和调整行政职权143项，率先在高新区推行“3550”高效集中审批改革试点。加强政务服务标准化建设，编制市级政务服务事项目录515项。“二十六证合一”等商事登记制度改革持续推进，各类市场主体增长18.1%。

稳步实施事业单位分类改革。优化整合、撤销调整12家事业单位，精简压缩市直及县区事业单位人员编制7032名。完成5家生产经营类事业单位改革任务。稳妥推进组建企业集团涉及事转企工作。

不断加快投融资体制改革。组建市产业（创业）投资引导基金，三生医疗、现代农业两支产业投资基金获省支持。大力推进华日高新材料、中科生物和长生生物新三板挂牌上市。引进光大银行等4家金融机构。银行业贷款余额突破1270.8亿元，比年初新增贷款194亿元，均创历史新高。

（三）统筹推进优环境，城乡面貌明显改善

大力实施主城区环境综合整治。拆除违建近5万处，自拆率95%以上，两年任务一年基本完成；整治老旧小区202个，改造街巷路146条，实施暖房子工程334万平方米，打造了长山社区、“二要小巷”、幸福彩云等一批样板工程，极大地改善了居民的生活条件。

积极推进美丽乡村建设。完成乡镇环境治理任务，创建美丽乡村示范村18个。加快特色乡镇培育，张其寨大翻身村成为全国生态文化村，二棚甸子镇入选全国特色小镇，草河掌镇、雅河乡等6个乡镇列入省级特色乡镇培育名单。

持续加强生态环境治理。营林造林13万亩、治理矿山195亩。全面推行了河长制，加强水源地污染治理。取缔10吨以下燃煤锅炉535台，淘汰黄标车老旧车辆3495台，空气质量优良天数318天。完成土壤环境质量监测网络建设。中央环保督察转办案件得到有效解决。

不断加快基础设施建设。辽中环高速公路本溪段、田桓铁路建设顺利推进，观音阁水库输水工程完成80%。改造维修解放路、溪湖路等城市道路15.2公里、农村公路187.3公里。彩屯大桥、鲍家两洞桥实现通车。高新区张仲景大街、太子河新城6条道路、威宁大街地下综合管廊等新城建设项目加快施工。智慧本溪建设迈出新步伐，“互联网+政务服务”获全国“十大最佳政务应用”等荣誉。

（四）多措并举惠民生，社会大局保持稳定

努力为群众办好事办实事。完成12234人脱贫、30个贫困村销号、桓仁县贫困县摘帽任务。城镇新增就业2.3万人，城镇登记失业率3.99%。棚户区改造开工8400多套，新增低收入住房保障家庭租赁补贴140户，筹集公租房实物房源260多套。义务教育均衡发展稳步推进，明山区通过国检、平山区通过省检。市中心医院新城院区门急诊开诊。企业退休人员养老金月人均增加130元、达到2362元，确保了按时足额发放。提高了社区工作者待遇标准。各县区城市低保标准分别提高到每月500元和550元，各县区农村低保标准均提高到每年4044元，实现了农村低保标准全市统一。拨付特困人员救助供养资金2502万元，免费为残疾人适配辅助器具2699件，完成贫困残疾人家庭无障碍改造700户。创建国家食品安全示范城市有序推进。新增民办养老机构16家、城乡社区养老服务设施和服务中心19个。

提高公共服务水平。启动第三期学前教育行动计划。建立城区义务教育联盟9个。完成东方剑桥本溪国际学校基础设施建设、太子河新城学校主体工程。以市中心医院、本钢总医院、市中医院为龙头，组建3个“医联体”。本溪湖工业遗产群入选国家建筑遗产名录和工业遗产名单。辽宁东北抗日义勇军纪念馆等文化项目建设稳步推进。反腐警示教育评剧《中秋泪》全省巡演，音舞诗画《映像本溪》获省艺术节金奖。开展全民健身活动，成功承办2016-2017赛季CBA联赛本溪主场等赛事。

促进社会和谐发展。深入开展“矛盾纠纷多元化解年”活动，坚持市长、县区长信访接待日制度，落实党政领导包案责任，化解信访积案647件。深化平安本溪、法治本溪建设，圆满完成重大维稳安保工作，安全生产、公共安全形势平稳，社会治安秩序良好。七五普法高起点开局、高标准推进。巩固双拥模范城创建成果，促进了军民融合发展。

此外，人防、外事、侨务、对台、民族、宗教、档案、史志、气象等工作也取得积极进展，妇女儿童、残疾人等事业得到新发展。

一年来，市政府不断加强自身建设。从严治党更加深入。推进“两学一做”学习教育常态化制度化，切实履行全面从严治党主体责任，理想信念进一步坚定。依法行政更加有力。认真办理市人大代表建议、政协提案470件。坚持规范行政执法行为，清理法规、规章、规范性文件35个。责任担当更加落实。建立亲清新型政商关系，以敢于担当的勇气、务实肯干的定力，推动经济社会持续健康发展。廉洁从政更加到位。严格落实中央八项规定，切实加强行政监察、审计监督，以公开公正、廉洁高效的政府形象凝聚起干事创业的强大力量。

过去五年，我们办成了一些多年来一直想办但没有办成的大事，办成了一些让老百姓有获得感的实事，办成了一些造福子孙后代的好事。本溪这五年的发展进步，得益于中央和省委、省政府的关怀指导，得益于市委的坚强领导，得益于市人大、市政协的监督支持，得益于全市广大干部群众的团结奋斗。在此，我代表市政府，向全市人民，向人大代表、政协委员，向各民主党派、工商联、无党派人士和各人民团体，向中省直单位、解放军驻溪部队和武警官兵，向所有关心支持本溪发展的各界人士，表示诚挚感谢和崇高敬意！

各位代表，回望这五年，我们有很多深刻的体会：做好政府工作，必须牢固树立“四个意识”，坚决维护以习近平同志为核心的党中央权威和集中统一领导，坚决落实市委决策部署;必须紧紧围绕发展第一要务，始终保持战略定力和发展耐心，一张蓝图绘到底，一任接着一任干；必须始终坚持以人民为中心，把保障和改善民生作为根本目的，让本溪人民生活富裕起来、幸福起来；必须切实强化改革意识、担当作为，面对困难不躲闪、不畏惧、不退缩，特别是在解决关乎城市发展、民生福祉的问题上，不怕得罪人，不怕有阻力，以动真碰硬、知难而进的勇气，以雷厉风行、久久为功的作风，诠释对党和本溪人民的忠诚！

同时，我们也清醒地看到，还存在一些问题：一是结构调整任务仍然艰巨，生物医药、钢铁精深加工、旅游产业发展规模不大、质量不够高，县域经济、产业园区、民营企业发展仍是短板，大项目少、好项目少、高层次人才少问题依然突出。二是营商环境迫切需要进一步优化，审批效率低等突出问题尚未根本解决。三是城市管理不到位，背街小巷脏乱差、交通拥堵、停车难、房地产开发不规范等问题亟待解决。四是财政收支矛盾越发突出，地方财力有限，刚性支出和养老金发放、政府债务偿还等压力增大。五是部分干部思想不解放、创新意识不强，不担当、不作为现象时有发生。我们将正视矛盾，正视困难，正视现实，采取有力措施加以解决。

二、今后五年奋斗目标和主要任务

未来五年，是深入贯彻党的十九大精神的关键时期，是全面深化改革的攻坚期，是全面建成小康社会的决胜期，全国各地将进入新一轮的发展热潮，中央关注支持东北振兴的政策还将继续出台，现在已经赋予辽宁近百个国家级战略平台，我省正在拉高标杆、奋起直追，明确了高质量发展的战略布局和目标任务。只要我们把这些政策用到最好、落到最实，本溪振兴发展就有扎实的后劲、十足的韧劲、强大的冲劲。对于我们本溪来说，今后五年注定充满机遇和希望。本溪自然资源丰富，产业基础雄厚，区位优势明显，开放前景广阔。尤其是这些年我们谋划了一批事关发展全局的大项目，建设了一批牵动作用强的好项目，储备了一批符合振兴政策导向的新项目，为未来高质量发展积蓄了后劲。本溪人民勤劳智慧、务实肯干，创造了坚实的发展基础。更为重要的是，本溪政治生态和从政环境良好，全市上下呈现出政通人和、风清气正、共谋发展的好局面。我们从来没有像现在这样，有信心和能力把本溪建设得更加令人留恋和向往，让本溪人民更加富裕、更加幸福、更有尊严地生活。我们有理由相信，本溪一定大有可为、大有希望，本溪的明天一定更美好！

今后五年总体要求

全面贯彻党的十九大精神，以习近平新时代中国特色社会主义思想为指导，坚持稳中求进工作总基调，坚持新发展理念和“四个着力”“三个推进”，牢牢抓住东北振兴“一带五基地”发展机遇，深度融入沈阳经济区建设，按照高质量发展要求，坚持以供给侧结构性改革为主线，坚定不移地实施工业强市、旅游兴市、生态立市发展战略，坚持一张蓝图绘到底，不断丰富和提升“三都五城”内涵，进一步做大做强绿色钢都、中国药都、枫叶之都，加快新城建设，疏解提升老城，促进乡村振兴，推动城市转型，统筹区域发展，保障改善民生，坚决打好防范化解重大风险、精准脱贫、污染防治攻坚战，促进经济繁荣、山川秀美、社会和谐，建设美丽健康幸福新本溪。

今后五年奋斗目标

到2020年，全面建成小康社会。全面完成“十三五”规划目标任务，全市五项主要经济指标增速高于全省平均水平1个百分点以上，县域经济综合实力排名逐年晋位升级，城乡居民收入比2010年翻一番，建档立卡贫困人口全面实现脱贫。到2022年，力争进入全省发展先进行列。全市五项主要经济指标增速高于全省平均水平2个百分点以上，绿色钢都、中国药都、枫叶之都建设成效显著，深度融入沈阳经济区建设取得重大进展，走出一条资源型城市高质量转型发展新路。

今后五年主要任务

——坚定工业强市不动摇。必须把实体经济作为实现高质量发展的着力点。巩固老优势，再造新优势。继续举全市之力支持本钢做优做强，推进提质增效、产品升级、智能制造、绿色发展，打造国内一流的精品板材基地。大力推进矿产资源集约开发利用，提高钢铁精深加工水平，加快产业园区建设，构建钢铁全产业链发展格局。坚定不移加快高新区建设发展，聚焦源头创新和产业升级，加大招商引资、招才引智力度，培育一批拥有自主知识产权、具有世界影响力的企业，把高新区打造成集医药、保健、养生、休闲为一体的健康新城。到2020年，钢铁深加工产业、生物医药产业占全市工业比重均达到8%。到2022年，钢铁深加工产业、生物医药产业占全市工业比重均达到15%，高新区力争进入国家高新区生物医药产业综合竞争力排名前20强。

——坚定旅游兴市不动摇。必须把旅游业作为拉动经济发展的重要动力。以创建全域旅游示范区为切入点，规划建设旅游产业集聚区，将本溪县、桓仁县打造成为知名国际旅游度假区。不断丰富枫叶之都品牌内涵，着力开发旅游主题产品，发展精品特色民宿等新业态。完善现代旅游公共服务体系建设，提高旅游景区通达性。组建本溪旅游集团，整合优质旅游资源，实现整体上市。到2020年，旅游收入增速高于沈阳经济区平均水平，把本溪建成沈阳经济区后花园。到2022年，5A级景区达到3个以上，将本溪建成全域旅游示范城市。

——坚定生态立市不动摇。必须树立和践行绿水青山就是金山银山的理念。坚决守住生态保护红线、永久基本农田、城镇开发边界三条控制线，全面推进蓝天、碧水、净土、农村环保和青山工程。全面落实污染防治与生态建设三年攻坚计划，加大对集中式饮用水水源地保护力度，建设全省生态保护屏障，森林覆盖率保持在76%以上，饮用水水质达标率100%，空气质量优良天数达到85%以上。到2020年，城乡生态环境明显改善。到2022年，建成生态文明城市。

——坚定开放创新不动摇。必须坚持引进来和走出去并重，加强创新能力开放合作。抢抓“一带一路”和东北老工业基地振兴机遇，聚焦“一带五基地”建设、“五大区域发展战略”，全方位融入沈阳经济区，主动承接沈阳辐射带动，把本溪建成沈阳经济区重要战略承载区及医药健康产业集聚地、高端装备制造业配套基地、旅游生活服务目的地、绿色生态共享地，构建功能互补、错位发展新格局。坚定不移优化营商环境，坚定不移招商引资上项目，大力实施创新“四项工程”。到2020年，对外开放水平、科技创新实力大幅跃升。到2022年，融入沈阳经济区发展取得突破，研发经费支出占地区生产总值比重达到2.5%以上，高新技术产品增加值年均增长15%以上。

——坚定发展县域经济不动摇。必须建立更加有效的区域协调发展新机制。统筹实施县域经济发展和乡村振兴战略。坚持新型工业化、农业现代化、新型城镇化三化同步，两县重点发展装备制造、健康食品、中药饮片等特色优势产业，“一村一品”实现行政村广覆盖，农产品深加工集聚区全面升级。深入推进农村改革，壮大农村集体经济，加强农村基础设施建设，改善农民生产生活条件，建成一批产业发达、环境优美、人文气息浓厚的特色小镇，推动农业全面升级、农村全面进步、农民全面发展。到2020年，本溪县综合经济实力跃升到全省前15名，桓仁县综合经济实力跃升到全省前20名。到2022年，两县六项主要经济指标高于全省县域经济平均水平2个百分点,县域经济支撑力显著提升。

——坚定加强和改善民生不动摇。必须把人民对美好生活的向往作为奋斗目标。既尽力而为，又量力而行，让改革发展成果更多地惠及全市人民。提升城市建设品质，围绕完善功能、丰富内涵，加快太子河新城、高新区建设；突出生态宜居功能、增强文化功能，加快老城疏解提升，促进太子河“一河居中、两岸繁荣”发展，把本溪打造得更加美丽、更加宜居。始终保持城乡居民收入增长与经济增长同步。努力让就业更加充分、社会保障更加优质。促进教育、卫生、文化、体育等社会事业全面进步。推进文化名市和文化名县建设。到2020年，全面建成小康社会。到2022年，全市人民日益增长的美好生活需要不断得到满足。

三、2018年工作安排

2018年是贯彻党的十九大精神的开局之年，是改革开放40周年，是决胜全面建成小康社会、实施“十三五”规划承上启下的关键一年。

按照市委经济工作会议部署，确定主要预期目标：地区生产总值增长6.5%，一般公共预算收入增长6%，固定资产投资增长10%，规模以上工业增加值增长7.5%，社会消费品零售总额增长5%，进出口总额占全省的份额不减，城乡居民收入增长与经济增长保持同步。

重点做好九个方面工作：

（一）加快高新区建设步伐

培育优质项目和企业。扶持一批具有核心竞争力的骨干企业，支持上海绿谷、恒康制药、上药集团、华润三药、天士力等企业优质产能加快释放。全力推进顺势产业园、杏林医疗美容产业园、鼎持生物等重点项目加快建设。支持药联制药、爱尔创等企业筹备主板上市，力争新增3户企业在新三板挂牌。

促进科技成果转化。搭建政府、企业、大学合作平台，发挥沈阳药科大学等高校和科研机构支撑能力，加速科技成果在高新区转化。集约高效利用现有土地资源，启动二期标准化工业厂房建设，引进孵化一批科技型中小微企业。

持续完善城市功能。完成高新区规划调整，不断加大基础设施建设和城市功能配套力度，打造全链条优质教育，进一步提升人气，建设宜居宜业的生态新城。

（二）推进工业经济增长

加快发展钢铁深加工产业。支持本钢一冷轧、三冷轧、高速线材等技改项目实施，推动四冷轧前期工作。发展壮大桥北、本溪湖、南芬经济开发区，重点引进汽车配件、五金制品等钢铁深加工项目。

大力发展民营经济。实施“大做强”工程，重点培育龙宝参茸、康顿门业、聚鑫达、宇航矿业等一批主业突出、核心竞争力强的民营龙头企业，力争新增销售收入亿元以上企业5户。实施“小升规”企业培育计划，精准帮扶100户成长型企业，实现“小升规”10户以上。

全面推进科技创新。重点在钢铁深加工、生物医药等领域，建设一批科技创新平台。新增高新技术企业5户，实现科技成果转化35项。引进培养科技领军人才、高层次技术人才，弘扬劳模精神和工匠精神，建设创新型、技能型劳动者队伍。

（三）推动旅游业提档升级

深化创建全域旅游示范区。规划建设两县国际旅游度假区，大力开发主题产品，支持关门山、五女山创建5A级景区，天著温泉中心、汤沟滑雪场、同泉滑雪场、枫林谷房车露营基地、大冰沟景区等项目建成运营。推广龙道沟民营股份制沟域旅游发展模式。设立本溪旅游产业基金，水洞旅游集团实现新三板挂牌。

加强旅游基础设施建设。加大旅游商品、特色美食开发力度，加快精品民宿、旅游公路和标准化旅游公厕建设，集本线三架岭隧道、本宽线北大岭隧道及引线工程建成通车。

强化旅游市场营销。深度开拓京津冀、东三省等客源市场，加强红色旅游遗址遗迹开发，筹划推出“重走抗联路”红色旅游、工业文化旅游、冬季旅游精品线路，塑造本溪旅游新形象。

（四）加快县域经济发展

推进农业结构优化升级。深化农村土地制度改革，落实北药产业发展规划，大力发展设施蔬菜、中药材、食用菌等特色高效农业，开展全域有机农业试验示范，指导调减低效益玉米种植面积。确保粮食安全。

大力发展特色产业园区。做强两县装备制造、玻璃制品、健康食品、中药饮片等特色优势产业，提升桓仁县农产品加工示范集聚区建设水平，本溪县尽快建成1个省级农业园区。扶持农业田园综合体建设，支持同仁堂、天士力参茸等9个产业融合发展试点项目建设。创立桓仁冰酒产品质量标准，打造冰酒特色品牌。扶持农业合作社和龙头企业发展，市级龙头企业稳定在120户左右。

推动新型城镇化建设。统筹规划部署乡村振兴与新型城镇化，切实加大财政投入力度，支持村集体经济发展和美丽乡村建设。推进两县国家级电子商务进农村综合示范县建设，重点实施本溪县电商产业园二期工程。提升益农信息社等综合服务水平。

（五）全力抓招商上项目

举全市之力抓招商。树立项目为王意识，充分发扬“五皮”精神，盯住长三角、珠三角和京津冀重点区域，聚焦“一带一路”、沈阳经济区、与江苏泰州合作等重要平台，主动“走出去、请进来”，争取更多优质企业、大项目落户本溪。

全力以赴加快项目建设。深入落实市、县区领导包扶项目建设制度，重点推进160个工业项目、71个服务业项目、70个基础设施项目，协调解决突出问题，确保项目按时间节点推进。加快推进福耀玻璃、思山岭铁矿、观音阁水库输水工程、万达广场、金地广场、红星美凯龙、兴隆大家庭、长江农副产品智能交易中心等一批在建项目早日竣工投产运营，天士力健康食品产业园、东软熙康云舍、本溪港桥北物流园、台湾永昕生物医药等一批签约项目开工建设，大力推进国电投氢能产业园、大台沟铁矿、桓仁抽水蓄能电站等一批项目加快前期工作，再谋划储备一批大项目、好项目，激发民间投资活力，增强发展后劲。

全力争取政策资金支持。积极争取产业转型升级示范区、民营经济试点等国家政策，推动田师付独立工矿区改造搬迁试点，包装申报新兴产业三年行动计划等项目。确保实现全年向上争取资金规模至少比2017年增长10%以上、各口径资金额度高于全省平均水平的目标。

狠抓优化营商环境。深入推进优化营商环境建设年活动，坚决执行“八个凡是”“七个不允许”，进一步加强营商环境评议和绩效考核工作，深入开展专项整治行动，严肃案件查办和责任追究。牢固树立与企业共命运理念，激发企业家精神，保护企业家权益，对纳税大户给予奖励，以最好的营商环境，让项目加快建设，让企业安心发展。

（六）深度融入沈阳经济区

积极参与制定沈阳经济区协同发展规划，在园区管理改革、生物医药产业聚集等方面取得突破。支持本钢、福耀玻璃、华岳精工等重点企业与宝马、通用、华晨对接合作，推动汽车及零部件产业协同发展。充分利用“沈阳港”引领作用，做大钢铁、医药等物流产业。积极争取10亿元产业（创业）引导基金。力争高新区与沈阳自贸区、沈抚新区政策协同，打造对外开放、创新发展政策高地。同时，促进与泰州市旅游资源互换、园区结对、企业交流，实现双赢互利发展。

（七）深化基础性关键性改革

深入推进“三去一降一补”。全面落实去产能、去库存、去杠杆、降成本、补短板各项任务，不断提高实体经济发展质量。

稳步推进机构和行政体制改革。深化“放管服”改革，进一步向高新区下放行政权力，深入开展“3550”改革试点取得成功经验全市推广。持续推进商事制度改革，激发市场主体活力。完成“市民之家”建设，真正实现“一站式”服务。全面实行并联审批，网上政务服务实现全覆盖。完成事业单位分类改革。

加快推进国有企业改革。推进国有企业混合所有制改革。完善市属重点国有企业 法人治理结构，实现市场化运营。对完成破产财产分配企业，加快破产终结。努力推动工业类“僵尸企业”处置，积极做好国有企业“三供一业”分离移交，推进国家厂办大集体改革政策落实，稳妥解决国企转制遗留问题。

深化财税体制改革。全面实施预算绩效管理，强化预算支出责任和效率，建立完整的绩效评价指标体系。推进乡镇财政体制改革。大力培植财源，依法组织税费收入，积极落实各项税收优惠政策，进一步减轻企业负担。

加快投融资体制改革。大力支持华岳精工等企业上市，华日高新材料等企业实现新三板挂牌。加快推进市商业银行、市区农信社改革，力争市商业银行更名为“本溪银行”。积极引进金融机构，加大金融服务实体经济力度，全年新增社会融资220亿元。

（八）全面提升城市治理水平

大力提升城市规划约束力。启动新一轮城市总规修编工作。强化规委会决策机制，严格规划审批，加强规划执行刚性控制，加大对未批先建等违法行为的打击力度，切实维护城乡规划权威性和严肃性。

加大基础设施建设力度。开工建设太子河公园，完善太子河“一河两岸”景观带。建设站前游园等10个城市公园和广场、25座主城区公厕。新建改造胜利路、文化路等城市道路，改造危桥2座。进一步完善太子河新城实施规划，强力推进征迁工作，加快道路、太子河新城学校等基础设施项目建设，积极引进五星级酒店、大型商业综合体项目。推进“新溪湖·印象”建设，进一步调整完善规划，加快基础设施建设和周边环境打造。推进沈本线响山子至滨河南路改扩建二期工程建设，力争年底建成通车。启动90公里绕城公路建设。推进桓仁支线机场、本桓（宽甸）高速公路、溪田铁路提速改造、环卫和垃圾焚烧发电一体化等项目前期工作取得进展。田桓铁路建成通车。

开展城市管理年活动。建立城市管理长效机制，推进城市净化、绿化、亮化、美化。继续深入开展主城区环境综合整治，实施城市道路交通拥堵治理，完成114条街巷路改造、108个老旧小区整治，强化小区物业规范管理，引导居民及社会组织参与，治理城市“脏乱差堵”取得显著成效。推进生活垃圾分类处理。加强城市管理综合执法，构建服务为先、管理优化、执法规范的城市治理体系。规范房地产市场管理，开发项目用地严格执行“净地出让”、公开竞拍，中标后一次性缴齐土地出让金和各类配套费，项目封顶后发放预售许可证，决不允许再出现搬迁无法回迁、购房无法入住、房产证无法办理的情况。

坚决打好污染防治攻坚战。巩固并扩大中央环保督察成果，严格落实生态保护红线，深入实施蓝天、碧水、净土、农村环保和青山工程。完成大气污染治理任务。实施北沙河环境综合治理，确保出市断面水质达到IV类标准，完成观音阁水库保护区调整、市级工业园区水污染治理工作。开展重点行业企业污染地块情况调查，完成农用地土壤污染数据调查。大力推进农业农村环境综合治理。营林造林11万亩。推进领导干部自然资源资产离任审计。推动两县开展国家级生态县创建工作，生态环境质量总体改善。

（九）切实增进民生福祉

坚决打好精准脱贫攻坚战。巩固脱贫攻坚成果，大力实施医疗救助，防止因病致贫、因病返贫。继续实施“五个一批”工程，精准施策，实现全市3001人脱贫、17个贫困村销号。

全面加强社会保障。积极征缴筹措养老金，确保按时足额发放。城乡低保标准平均提高5%以上，特困供养标准按照低保标准比例同时上调。城镇新增就业2万人，城镇登记失业率控制在4.5%以内，保持就业形势总体稳定。提高全市最低工资标准。改造棚户区4803套，新增低收入住房保障家庭租赁补贴100户，实现最低收入群体住房租赁补贴全覆盖。为500户贫困残疾人家庭进行无障碍设施改造。不断提高养老服务质量，全力推进失能老人养护楼项目建设。

稳步发展社会事业。全面提升教育教学品质，加强师德师风建设，7个县区义务教育均衡发展全部通过国检，加快辽宁中医药大学本溪校区等项目建设，辽宁科技学院产教融合项目投入使用。全力推进文化事业和文化产业发展，加快市人民文化宫舞台综合体建设，加快推进本溪湖工业遗产群一号高炉保护修缮工程，全面铺开基层综合性文化服务中心建设，办好群众文化节。推进健康本溪建设，继续深化公立医院综合改革，加大全科医生培训力度，完善分级诊疗制度，开展紧密型医疗联合体建设试点工作。大力开展全民健身活动，不断完善城市体育公园和健身场地建设，承办好CBA本溪主场赛事，做好申办2024年冬运会分赛场工作。大力弘扬本溪精神，建设诚信友善本溪。

保持社会平安稳定。加强社会治安防控体系建设，把“民生警务”理念融入执法活动全过程，满足人民群众对公平正义的新期待，从严打击各类违法犯罪、从严社会面管控、从严公共安全管理，不断提升人民群众的幸福感、获得感和安全感。严格落实领导干部接访下访制度，切实化解一批信访积案。全面提升安全生产监管水平，确保不发生重特大安全生产事故。加强食品药品安全监管，保障百姓饮食用药安全。实施七五普法规划，全方位推进公共法律服务体系建设，推动食品检验中心尽快投入使用，推行食用农产品市场准入制度。继续深化双拥共建，促进军民融合发展。全面做好人防、外事、侨务、对台、民族、宗教、档案、史志、气象和机关事务等工作。

四、建设人民满意政府

新时代要有新气象，更要有新作为。加快本溪振兴发展，全面建成小康社会，要求我们必须加强政府自身建设，必须从严治政，建设忠诚担当、公开公正、清正廉洁、诚信务实的人民政府。

旗帜鲜明讲政治。把党的建设摆在首位，全面推进从严治党，扎实开展“不忘初心、牢记使命”主题教育，深入学习贯彻党的十九大精神，坚持用习近平新时代中国特色社会主义思想武装头脑、推动工作，坚决维护以习近平同志为核心的党中央权威和集中统一领导，坚决贯彻中央和省委市委决策部署，不折不扣落实到位、执行到位。

全面加强依法行政。严格执行政府重大事项集体决策制度，完善土地出让、城市管理、公共资源交易等工作规则，管住人、管住事、管住权。自觉接受人大的依法监督、政协的民主监督、社会和舆论监督，全力办好人大代表建议、政协提案，依法公正用权履职。

永葆清正廉洁本色。时刻将纪律和规矩挺在前面，坚决落实八项规定新实施细则，坚持不懈纠正“四风”，深入推进党风廉政建设和反腐败斗争，严厉惩治侵害群众利益的不正之风和腐败问题，建立清清爽爽的同志关系，努力营造风清气正的干事创业氛围，实现干部清正、政府廉洁。

求真务实担当作为。解放思想创新干，破除一切影响发展的思想障碍，摒弃对传统路径的依赖。只争朝夕加快干，跑出比别人更快的速度，干出比别人更高的效率。敢于担当迎难干，勇于接硬任务，善于啃硬骨头。勤勉敬业埋头干，不当评论员，要当实干家。狠抓落实，崇尚实干，干一件成一件、做一桩成一桩，绝不能懒政怠政、碌碌无为，让本溪的发展耽误在我们手里；绝不能好大喜功、沽名钓誉，造成遗憾和败笔，给后人留下烂摊子；绝不能寅吃卯粮、透支未来，砸了子孙后代的饭碗。

各位代表，让我们在市委的坚强领导下，紧紧依靠和团结带领全市人民，不忘初心，牢记使命，奋进新时代，开启新征程，为全面建成小康社会，为加快建设美丽健康幸福新本溪而努力奋斗！
